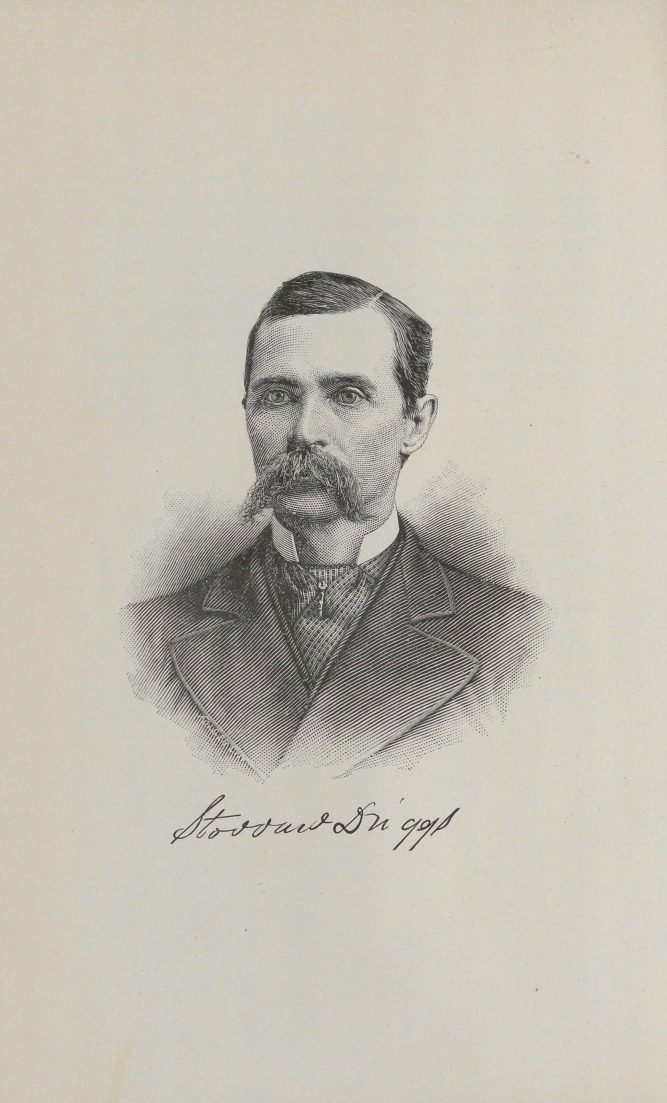# Obituary

**Published:** 1888-10

**Authors:** 


### Obituary.

Dr. Stoddard Driggs, D.D.S., of Lexington, Ky., was born at Rome, New York, January 1st, 1828, and died in the city of Lexington, Ky., July 18th, 1888.

He began the practice of his profession in the town of Auburn, New York, he remained there but a short time when he removed to Georgetown, Kentucky, where he practiced about two years, and then returned to his native place, and remained there some months; after which he returned to Kentucky and located in the city of Lexington, which proved to be the field of his professional career till the time of his death.

He was engaged in active practice till within a few weeks of his death. Dr. Driggs was a graduate of the Ohio College of Dental Surgery having received the degree of D.D.S., in 1862.

He was a man of much more than ordinary skill in his profession; ever trying to keep abreast of the current of progress in his chosen field, and never failing to give to those committed to his care the benefit of his best efforts.

In his day few men were his equal, and doubtless no one his superior in the conduct of office practice; indeed it can be said that his life was a rhythm of punctuality, the embodiment of just intentions and sympathetic assurance to his patients.

He was no friend of shams or frauds, but gave to every man the recognition he deserved; honest in word and deed himself, he could not brook dishonest work or words in others.

His charity, though frequent and to the full measure of his circumstances was like his life, always unostentatious. To all young men of our profession with whom he came in contact, he was ready to lend a helping hand, especially if he was convinced that they were ready to help themselves.

Dr. Driggs in his professional, social, and moral life was an exemplar of true goodness, a man among men, commanding the respect and love of all who knew him. The breach made by the removal of such men as Dr. Driggs from the profession is not easily filled. The honor and distinction he achieved should be a stimulus to all young men of the profession to emulate the noble example so clearly set before them. The portrait of Dr. Driggs in this number of the Register is an excellent likeness and will be appreciated by all who knew him. R.

---

Removing Indelible Ink.—Physicians are often asked how to remove idelible ink, and they sometimes cannot quite remember; so we repeat the following method: First moisten the stain with tincture of iodine, and after a few minutes, remove the iodine stain with solution of hyposulphite of soda. Finally wash in clean water. Repeat if necessary.

## Figures and Tables

**Figure f1:**